# Generating Full-Field Digital Mammogram From Digitized Screen-Film Mammogram for Breast Cancer Screening With High-Resolution Generative Adversarial Network

**DOI:** 10.3389/fonc.2022.868257

**Published:** 2022-04-29

**Authors:** Yuanpin Zhou, Jun Wei, Dongmei Wu, Yaqin Zhang

**Affiliations:** ^1^ School of Computer Science and Engineering, Sun Yat-sen University, Guangzhou, China; ^2^ Perception Vision Medical Technology Company Ltd., Guangzhou, China; ^3^ Department of Radiation Therapy, Nanxishan Hospital of Guangxi Zhuang Autonomous Region, Guilin, China; ^4^ Department of Radiology, Fifth Affiliated Hospital of Sun Yat-sen University, Guangzhou, Guangdong, China

**Keywords:** high resolution, conditional generative adversarial network, deep learning, breast cancer screening, mammography

## Abstract

**Purpose:**

Developing deep learning algorithms for breast cancer screening is limited due to the lack of labeled full-field digital mammograms (FFDMs). Since FFDM is a new technique that rose in recent decades and replaced digitized screen-film mammograms (DFM) as the main technique for breast cancer screening, most mammogram datasets were still stored in the form of DFM. A solution for developing deep learning algorithms based on FFDM while leveraging existing labeled DFM datasets is a generative algorithm that generates FFDM from DFM. Generating high-resolution FFDM from DFM remains a challenge due to the limitations of network capacity and lacking GPU memory.

**Method:**

In this study, we developed a deep-learning-based generative algorithm, HRGAN, to generate synthesized FFDM (SFFDM) from DFM. More importantly, our algorithm can keep the image resolution and details while using high-resolution DFM as input. Our model used FFDM and DFM for training. First, a sliding window was used to crop DFMs and FFDMs into 256 × 256 pixels patches. Second, the patches were divided into three categories (breast, background, and boundary) by breast masks. Patches from the DFM and FFDM datasets were paired as inputs for training our model where these paired patches should be sampled from the same category of the two different image sets. U-Net liked generators and modified discriminators with two-channels output, one channel for distinguishing real and SFFDMs and the other for representing a probability map for breast mask, were used in our algorithm. Last, a study was designed to evaluate the usefulness of HRGAN. A mass segmentation task and a calcification detection task were included in the study.

**Results:**

Two public mammography datasets, the CBIS-DDSM dataset and the INbreast dataset, were included in our experiment. The CBIS-DDSM dataset includes 753 calcification cases and 891 mass cases with verified pathology information, resulting in a total of 3568 DFMs. The INbreast dataset contains a total of 410 FFDMs with annotations of masses, calcifications, asymmetries, and distortions. There were 1784 DFMs and 205 FFDM randomly selected as Dataset A. The remaining DFMs from the CBIS-DDSM dataset were selected as Dataset B. The remaining FFDMs from the INbreast dataset were selected as Dataset C. All DFMs and FFDMs were normalized to 100*μm* × 100*μm* in our experiments. A study with a mass segmentation task and a calcification detection task was performed to evaluate the usefulness of HRGAN.

**Conclusions:**

The proposed HRGAN can generate high-resolution SFFDMs from DFMs. Extensive experiments showed the SFFDMs were able to help improve the performance of deep-learning-based algorithms for breast cancer screening on DFM when the size of the training dataset is small.

## 1 Introduction

Breast cancer has become one of the leading causes of cancer death in women (1). It is crucial to detect breast cancer in the early stages because early detection leads to a higher survival rate (2). Mammography screening is one of the most effective methods for the early diagnosis of breast cancer. Previous studies show that mammography screening reduces the mortality rate of breast cancer (3–7).

Digitized screen-film mammography (DFM) and full-field digital mammography (FFDM) are two major techniques for mammography screening. Although FFDM has become the standard procedure for breast cancer screening, DFM had been widely used and well-studied in the past. Leveraging the well-studied DFM for better breast cancer screening in FFDM has become a vital topic for developing a better breast cancer screening system. Previous studies found that FFDM and DFM have no significant difference in cancer detection rate other than visual differences (8, 9). In this paper, we proposed to close the gap between FFDM and DFM with a high-resolution generative algorithm.

With the rapid development of deep learning algorithms, deep-learning-based computer-aided diagnosis (CAD) systems have shown significant potential in automatic breast cancer screening (10, 11). However, the application of deep-learning-based CAD systems is limited due to the lack of labeled data since well-annotated medical images are difficult and laborious to acquire. In the case of breast cancer screening with mammography, large-scale public FFDM datasets with mass and calcification annotations are yet to be built. Most FFDM CAD systems are built based on limited size in-house datasets. Fortunately, large-scale DFM datasets with annotations (12) are available publicly, yet utilizing these DFM datasets for building better FFDM CAD systems remains a vital challenge.

Conditional generative adversarial network (cGAN) (13) algorithms, including Pix2pix (14), pix2pixHD (15), and Cycle-GAN (16), have been particularly successful in image-to-image translation. Additionally, Cycle-GAN is state-of-the-art for unsupervised image translation. However, Cycle-GAN is not ideal for high-resolution image-to-image translation while other high-resolution image-to-image translation methods such as Pix2pixHD require supervised training with paired datasets.

In this study, we proposed HRGAN to tackle the challenge of leveraging DFM for building better FFDM CAD systems by closing the gap between DFM and FFDM with a generative algorithm. Moreover, our proposed HRGAN required no additional annotation, which makes it easy to apply to existing FFDM CAD systems. Our method is based on the unsupervised image translation algorithm Cycle-GAN. To generate high-resolution FFDM from DFM, a pair with constraint (PWC) training strategy was purposed. Additionally, multi-scale networks were purposed in our method to better capture details such as mass boundary and micro-calcifications. We further evaluate our method in two breast cancer screening tasks. Extensive experiments showed the synthesized FFDMs (SFFDMs) generated by HRGAN were able to help improve the performance of deep-learning-based algorithms for breast cancer screening on FFDM when the size of the training dataset is small.

This work is a further development based on our preliminary work (17). The present work complements the preliminary one in several aspects. First, we improve the discriminators by introducing the gradient map as input, which is inspired by GGGAN (18), a recent study for generating FFDM from digital breast tomosynthesis (DBT). Second, extensive experiments, including the mass segmentation and micro-calcification detection tasks, were conducted while the preliminary one only evaluated with the density estimation task. Moreover, we present a more in-depth discussion and analysis of the proposed method.

## 2 Materials and Methods

This section is organized as below. We first describe the data we used in our study. Second, the overall architecture of HRGAN is presented. Last, we present detailed information on the essential components of HRGAN in the following subsections.

### 2.1 Screening Mammography Data

Two public screening mammography datasets, a DFM dataset CBIS-DDSM (19, 20) and a FFDM dataset INbreast (21), were included in our study. The CBIS-DDSM, namely Curated Breast Imaging Subset of DDSM, is an updated and standardized version of the Digital Database for Screening Mammography (DDSM). While DDSM was a large-scale screening mammography dataset containing 2620 mammography studies, a relatively large subset was selected from DDSM making CBIS-DDSM still a large-scale DFM dataset. The CBIS-DDSM dataset includes 3568 DFMs with verified pathology information. The INbreast dataset has a total of 410 FFDMs with annotations of masses, calcifications, asymmetries, and distortions.

The above two mammography datasets were then recombined into three independent datasets for this study. There were 1784 DFMs from CBIS-DDSM and 205 FFDMs from INbreast randomly selected into Dataset A. Mammograms belonging to the same patient should be selected together during the random selection process. The remaining DFMs in the CBIS-DDSM dataset were selected as Dataset B. The remaining FFDMs in the INbreast dataset were selected as Dataset C. All mammograms were resampled to an isotropic pixel resolution of 100*μm* × 100*μm*. Patches for training HRGAN were cropped from the resampled mammograms. The size of patches was set to be 256 × 256 pixels in our experiment.

### 2.2 The Proposed HRGAN

**Figure 1:**
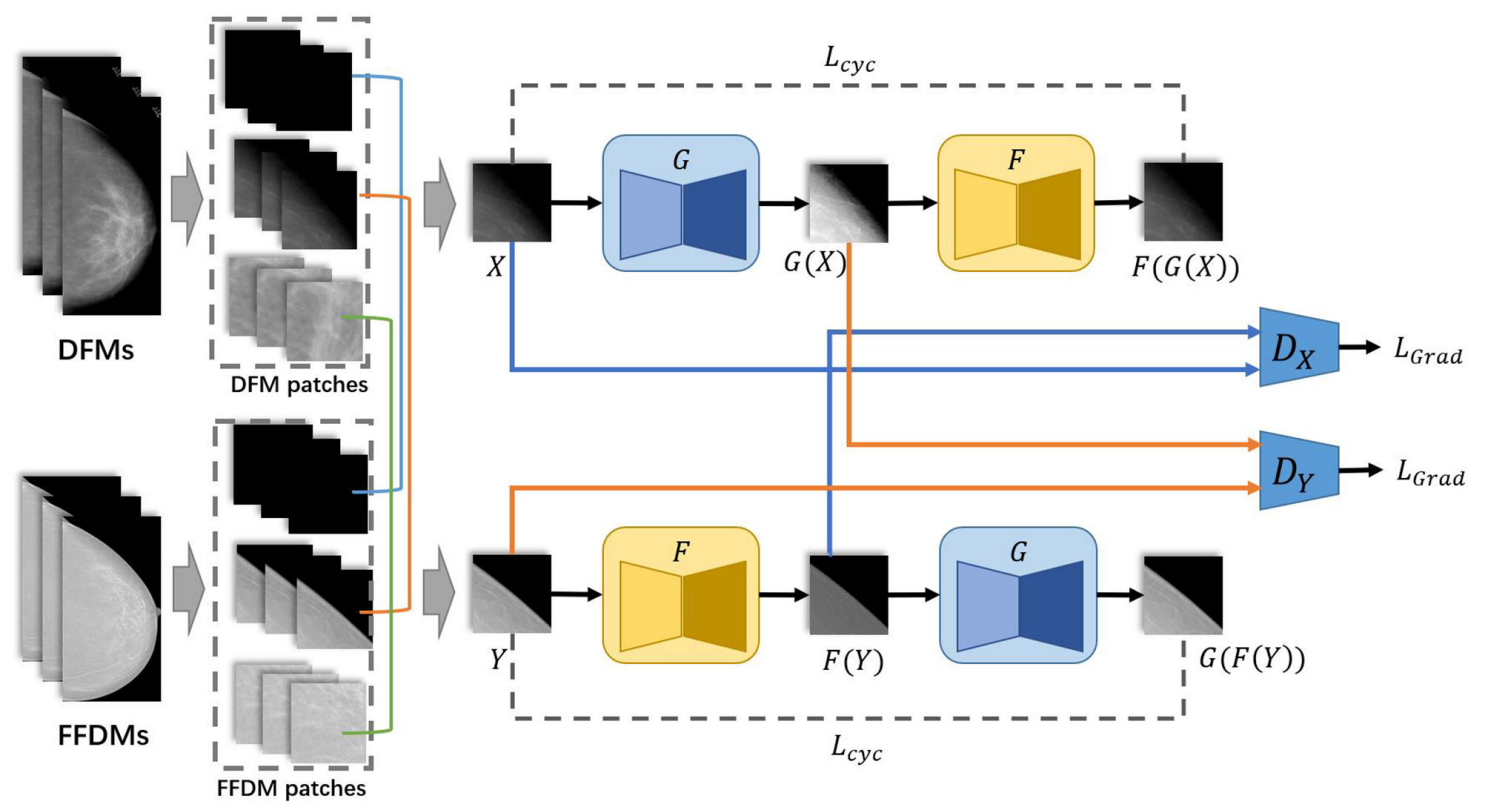
Overall architecture of HRGAN.

The overall architecture is shown in **Figure 1**. First, DFMs and FFDMs were cropped into small patches with the sliding window method. The threshold method OTSU (22) was applied to extract the background of mammograms. Patches were assigned to the categories of breast region, boundary, or background depending on the ratio of background in the patches. Second, these patches were used as input of HRGAN. However, unlike the vanilla Cycle-GAN where the input is a pair of images randomly picked from the two objective domains, we applied the pair with constraint (PWC) training strategy where the input pair is picked from the same categories of the two objective domains. We used U-Net (23) as the generators and a multiscale DNN architecture (15) as the discriminators. More details are described in the following subsections. In the inference stage, the trained generator was applied to DFMs to generate synthetic FFDMs (SFFDMs). Note that the model trained on patches can be applied to full-field screening mammograms because our generators were fully convolutional networks (24).

#### 2.2.1 The Pair With Constraint (PWC) Training Strategy

The PWC training strategy is simple but essential to our method. Before applying the PWC training strategy, all patches cropped from mammograms should be assigned to their corresponding categories. As described above, mammograms were first cropped into small patches by a sliding window. Second, the background in the patches was extracted by threshold methods and the percentages of background in the patches were calculated. If the whole patch was cropped from the background, then it is assigned to the background category. If no background is contained in the patch, it is assigned to the breast region category. The remaining patches were assigned to the boundary category.

The PWC training strategy was applied to select the input pair for training HRGAN after all patches were assigned to the three categories. First, a patch was randomly picked from all DFM patches. Its corresponding category (breast, boundary, or background) was marked. Second, another patch was randomly picked from the same category of FFDM patches. The selected DFM and FFDM patches formed the input of HRGAN, unlike Cycle-GAN where the input is a pair of images randomly picked from the two objective domains, resulting in a possible situation where a background patch could eventually be paired with a breast region patch as input and introduce noise to the training stage. The PWC training strategy simply divided the patches into three categories based on background percentages and paired patches only from the same category and eliminated noisy input from the model.

#### 2.2.2 The Network Architecture of The Generator

**Figure 2:**
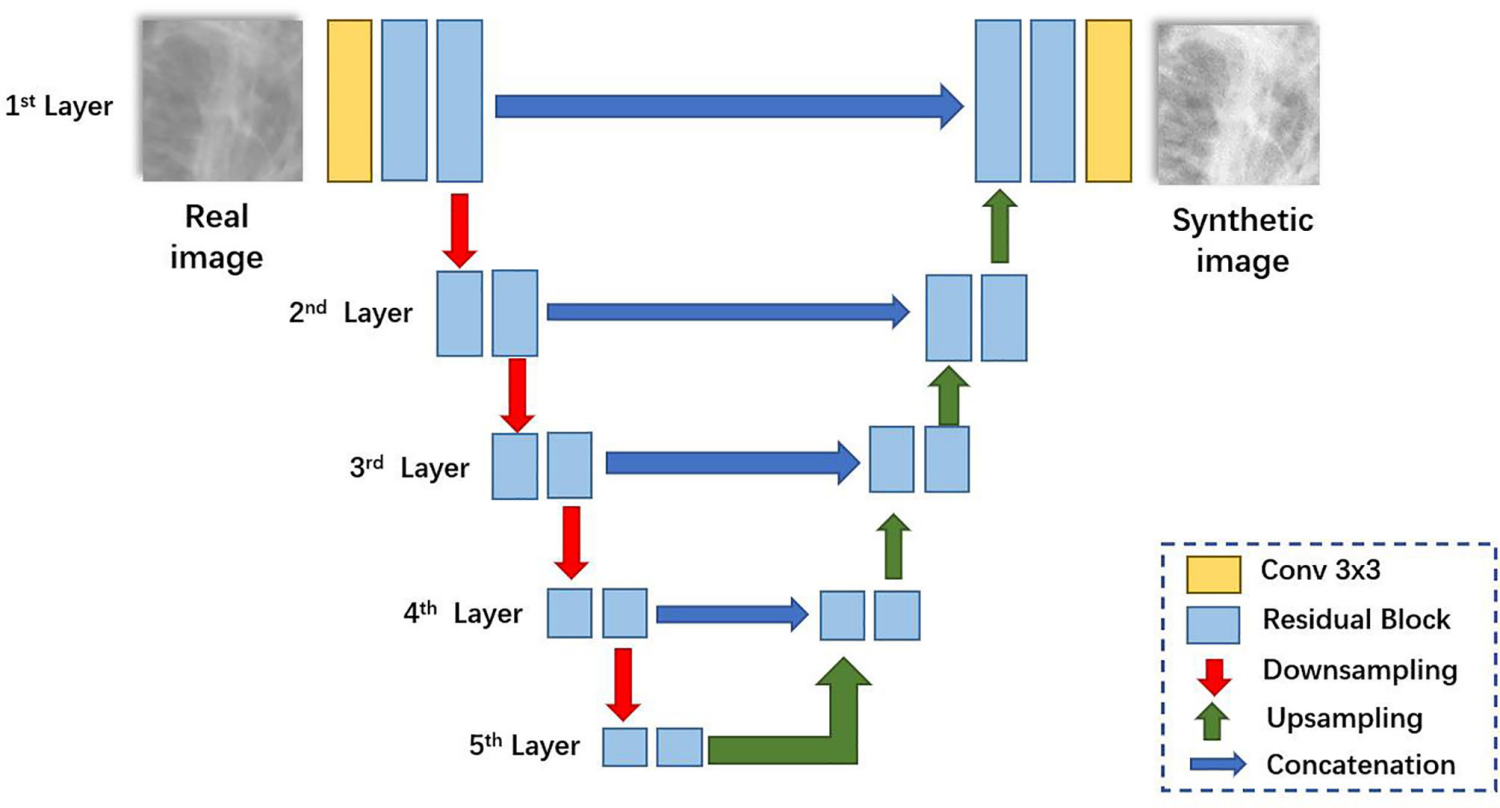
The network architecture of the generator.

The network architecture for generators is illustrated in **Figure 2**. Like U-Net (23), it consists of a contracting path (left side) and an expansive path (right side). First, the input image is fed into a convolutional block to extract low-level feature maps. The features are then fed through residual blocks (25) to extract higher-level feature maps. Then the feature maps are downsampled and fed into the next layer. The contracting path and the expansive path follow the typical architecture of a convolutional network. Skip connections (23) are applied to each layer to concatenate features of each layer in contracting patches with features in the expansive path.

#### 2.2.3 The Network Architecture of The Discriminator

**Figure 3:**
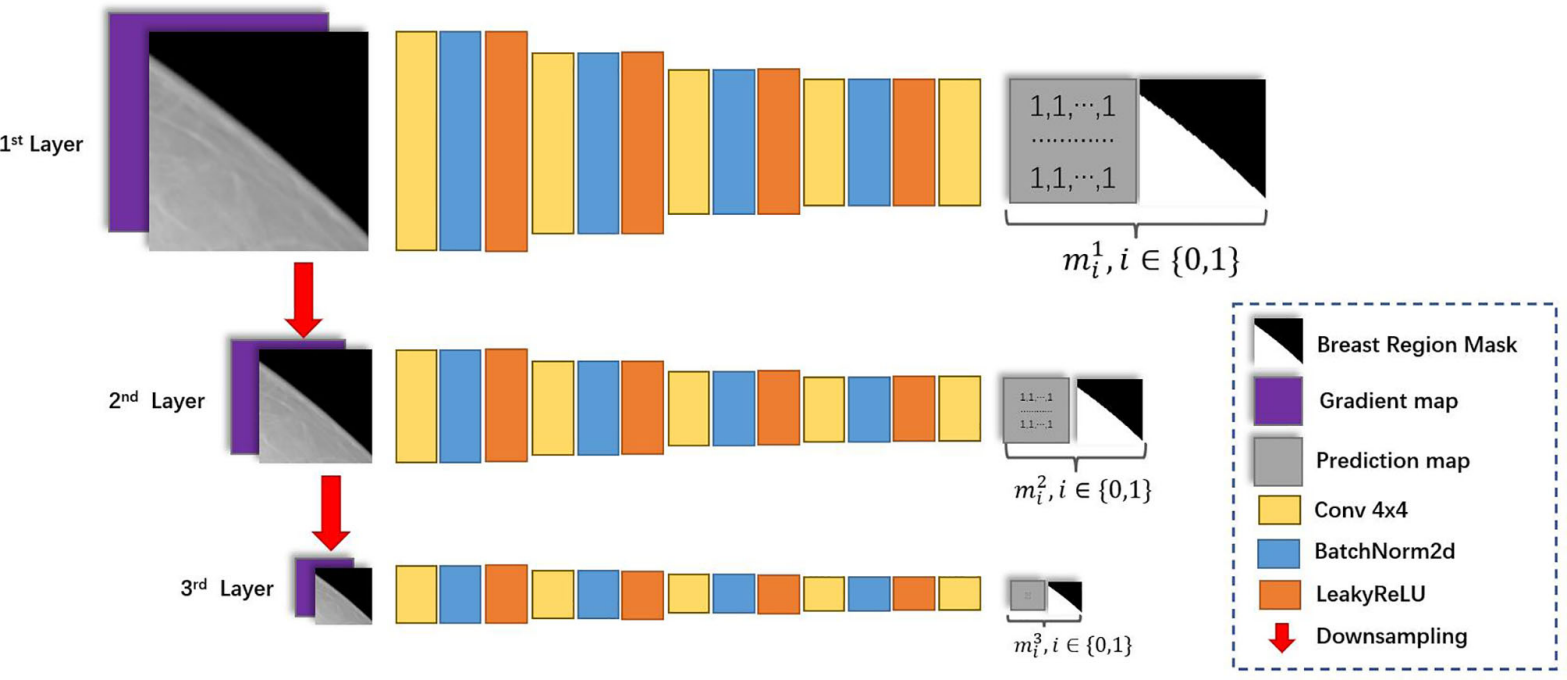
The network architecture of the discriminator.

The network architecture for discriminators is illustrated in **Figure 3**. Inspired by Pix2pixHD (15), we applied the multi-scale discriminator architecture in Pix2pixHD to HRGAN. Additionally, we modified the input and output of the multi-scale discriminator to better distinguish subtle differences between real and synthesized images. First, the gradient map of the input image is calculated through the Sobel filter (26). The input image as well as its corresponding gradient map were concatenated and fed through the first layer. Second, the input image is downsampled and its corresponding gradient map is calculated. The concatenation of the downsampled image and its corresponding gradient map is fed through the second layer. We denoted the input image as *X* and its corresponding gradient map as *X'*. Then the input for the *l*-th layer of the discriminator can be formulated as


(1) $$X_{\text{l}}=[X_{\frac{1}{2^{\left(l-1\right)}}},(X_{\frac{1}{2^{\left(l-1\right)}}}{)}^{\prime }],l\in \{1,2,3\}$$

where $X_{\frac{1}{2^{\left(l-1\right)}}}$ denoted *X* downsampled with factor *2* ^(^ *^l^* ^-1)^.

Introducing the gradient map as additional input for the discriminator was inspired by GGGAN [19], a recent work that was proposed to generate SFFDM from DBT. The study has shown that introducing the gradient map enhanced the weak edges to preserve small-scale structures such as subtle micro-calcifications in SFFDM. Additionally, we found signing discriminators with breast region boundary segmentation task helped discriminators better distinguish synthesis mammograms from real mammograms. Hence, the output is a two-channel map, where the first channel is the prediction map, and the second channel is a downsampled segmentation map indicating the breast region for the input image. We denoted II as a matrix whose value of every element is 1 with the same size of the downsampled segmentation map, *m^l^* as the downsampled segmentation map of the *l*th layer, and [∙,∙] as the concatenate operation. The output of the *l*-th layer of the discriminator can be formulated as


(2) $$m_{\text{i}}^{\text{l}}=[i\times I,m^{l}],i\in \left\{0,1\right\},l\in \left\{1,2,3\right\}$$

By forcing the discriminator to do the breast segmentation task, we implicitly guide the generators to learn the difference between the inside and the outside of the breast region.

#### 2.2.4 Loss Functions for HRGAN

We denoted *X* as a selected patch from DFMs, *Y* as a selected patch from FFDMs, *G*: DFM→FFDM and *F*: FFDM→DFM as generators, *D_X_* as the multi-scale discriminator to distinguish real and synthesized DFMs, and *D_Y_* as the multi-scale discriminator to distinguish real and synthesized FFDMs. Additionally, we denoted $\hat{X}=F(Y)$ and $\hat{Y}=G(X)$ . 

The loss function for backpropagating discriminator *D_X_* can be formulated as 


(3) $$L_{\text{Grad}}(D_{X})=\sum_{l=1}^{3}\left[{\left(D_{X}^{l}(X_{\text{l}})-m_{1}^{\text{l}}\right)}^{2}+{\left(D_{X}^{l}({\hat{X}}_{l})-m_{0}^{\text{l}}\right)}^{2}\right]$$

where $D_{X}^{l}$ is denoted the *l*-th layer of the multi-scale discriminator *D_X_* and ${\hat{X}}_{l}=[{\hat{X}}_{\frac{1}{2^{\left(l-1\right)}}},\;{\hat{X}}_{\frac{1}{2^{\left(l-1\right)}}}{)}^{\prime }]$ .

Similarly, we have


(4) $$L_{\text{Grad}}(D_{Y})=\sum_{l=1}^{3}[(D_{Y}^{l}(Y_{\text{l}})-m_{1}^{\text{l}}{)}^{2}+{\left(D_{Y}^{l}({\hat{Y}}_{l})-m_{0}^{\text{l}}\right)}^{2}]$$

where $D_{Y}^{l}$ is denoted the *l*-th layer of the multi-scale discriminator *D_X_* and ${\hat{Y}}_{l}=[{\hat{Y}}_{\frac{1}{2^{\left(l-1\right)}}},{\hat{Y}}_{\frac{1}{2^{\left(l-1\right)}}}{)}^{\prime }]$ .

The loss function for backpropagating generator *G* follows Cycle-GAN, which can be formulated as


(5) $$L(G)=L_{\text{GAN}}(D_{Y},\hat{Y})+\lambda L_{\text{cyc}}(G,F,X)$$

where *λ* is the hyperparameter to balance *L*_GAN_ and *L*_cyc_


(6) $$L_{\text{GAN}}(D_{Y},\hat{Y})=\sum_{l=1}^{3}{\left(D_{Y}^{l}({\hat{Y}}_{l})-m_{1}^{l}\right)}^{2}$$

(7) $$L_{\text{cyc}}(G,F,X)=∥F(G(X))-X{∥}_{1}$$

Similarly, the loss function for backpropagating generator *F* follows Cycle-GAN, which can be formulated as


(8) $$L(F)=L_{\text{GAN}}(D_{X},\hat{X})+\lambda L_{\text{cyc}}(F,G,Y)$$

where *λ* is the hyperparameter mentioned above


(9) $$L_{\text{GAN}}(D_{X},\hat{X})=\sum_{l=1}^{3}{\left(D_{X}^{l}({\hat{X}}_{l})-m_{1}^{l}\right)}^{2}$$

(10) $$L_{\text{cyc}}(F,G,Y)=∥G(F(Y))-Y{∥}_{1}$$

The training procedure for HRGAN follows Cycle-GAN. At each iteration, generators are fixed and discriminators are updated. Then discriminators are fixed and generators are updated.

## 3 Experimental Results

This section is organized as follows. First, we describe detailed information on the experimental setup. Then we describe our evaluation metrics. Last, we present the experimental results.

### 3.1 Experimental Setup

As is described in Section 2.1, we used datasets A,B,C created from the CBIS-DDSM dataset and the INbreast dataset for our study. First, our proposed HRGAN was trained on dataset A. We set the hyperparameter λ = 10. We used Adam solver (27) with a batch size of 16. All networks were trained from scratch with a learning rate of 0.0005. We kept the same learning rate for the first 80 epochs and linearly decayed the rate to zero over the next 120 epochs. Second, SFFDMs were generated from dataset B by the HRGAN trained on dataset A. Third, two tasks for breast cancer screening, a mass segmentation task and a calcification detection task, were performed on dataset C. FFDMs on dataset C were downsampled to400*μm* for the segmentation task. The 100*μm* FFDMs on dataset C were tiled into 224 × 224 pixel-sized patches for the calcification detection task. Patches containing more than 80% of background were removed. Patches containing calcifications were given the label 1; otherwise, they were given the label 0. The goal of the calcification detection task is to classify these patches into two categories. U-Net (23) model was used for the segmentation task. Vgg-16 (28) was used for the calcification detection task.

Fivefold cross-validation (29) was performed on dataset C for the breast cancer screening tasks. For each fold, the U-Net and Vgg-16 models were trained on the training set of dataset C. They were denoted as the baseline models. We used Adam solver (27) with a batch size of 8 and a learning rate of 0.0001 for training the baseline U-Net. We used Adam solver (27) with a batch size of 16 and a learning rate of 0.0005 for training the baseline Vgg-16. To show the usefulness of HRGAN, we trained another U-Net model and another Vgg-16 model on the SFFDMs generated from dataset B. Similarly, we downsampled the SFFDMs to 400*μm* for the segmentation task and tiled the 100*μm* SFFDMs into 224 × 224 pixel-sized patches for the calcification detection task. Then we finetuned these two models on the training set of dataset C. We denoted them as the finetuned models. We used Adam solver (27) with a batch size of 8 and a learning rate of 0.0001 for training the finetuned U-Net. We used Adam solver (27) with a batch size of 16 and a learning rate of 0.0005 for training the finetuned Vgg-16. We set the learning rate to 0.00005 for both finetuned models at the finetuning stage and finetuned them for 200 epochs.

### 3.2 Evaluation Metrics

We used dice coefficient to evaluate the segmentation task. The dice score can be formulated as


(11) $$\text{dice}=\frac{2\left|A\cap^{}B\right|}{\left|A\right|+\left|B\right|}$$

Here *A* is denoted as ground truth, *B* is denoted as the prediction.

For the calcification detection tasks, we used the area under the receiving operator characteristic (ROC) curve (AUC) (30) to evaluate the performance of the classification models.

### 3.3 Results

**Figure 4:**
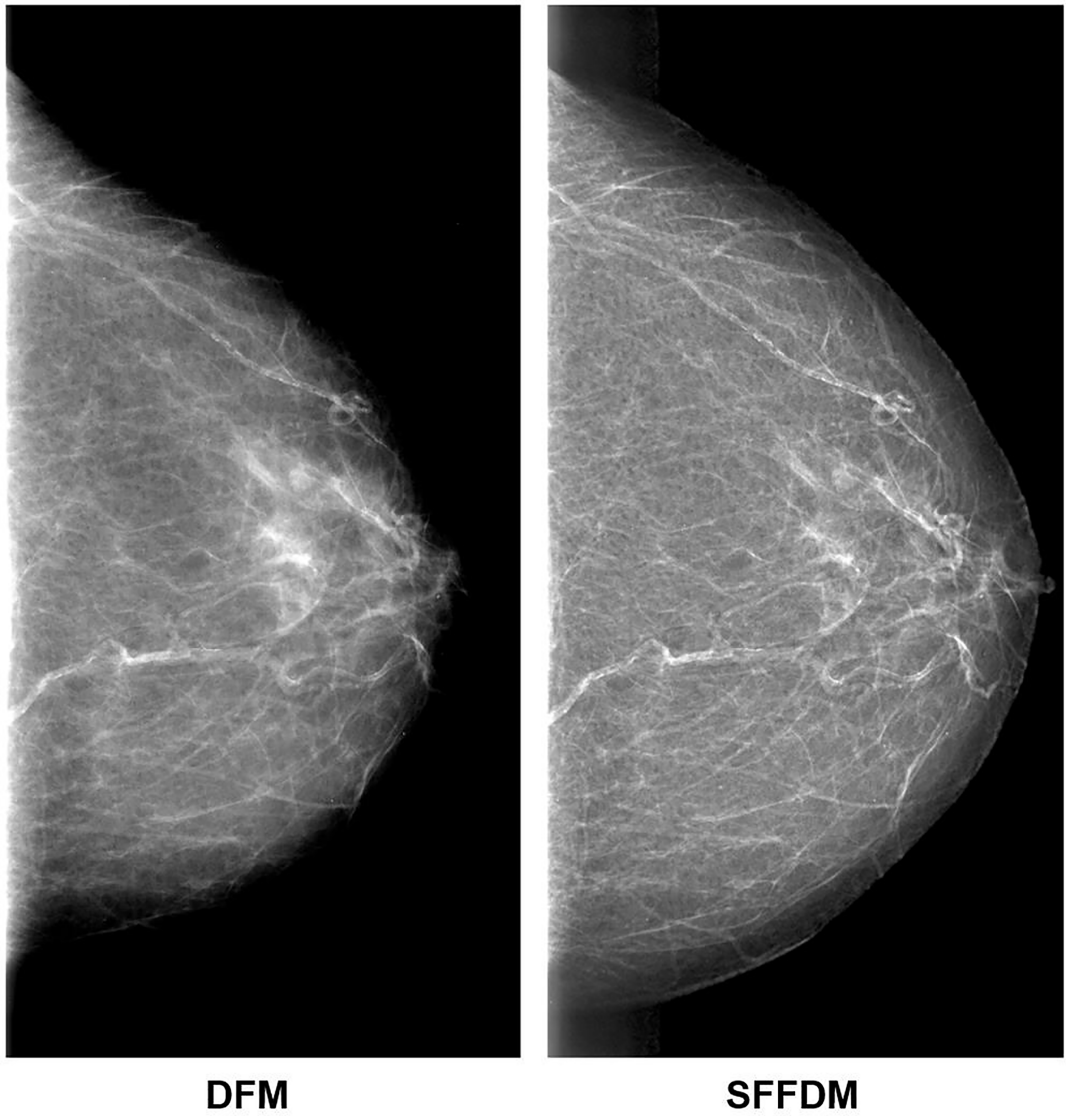
Visual comparison between DFM and SFFDM. Breast tissues are enhanced in SFFDM compared to DFM. Additionally, the breast region boundary was barely visible in the left DFM while the boundary was complete and clear in the right SFFDM. This clear boundary helped us locate the nipple position easily.

**Figure 5:**
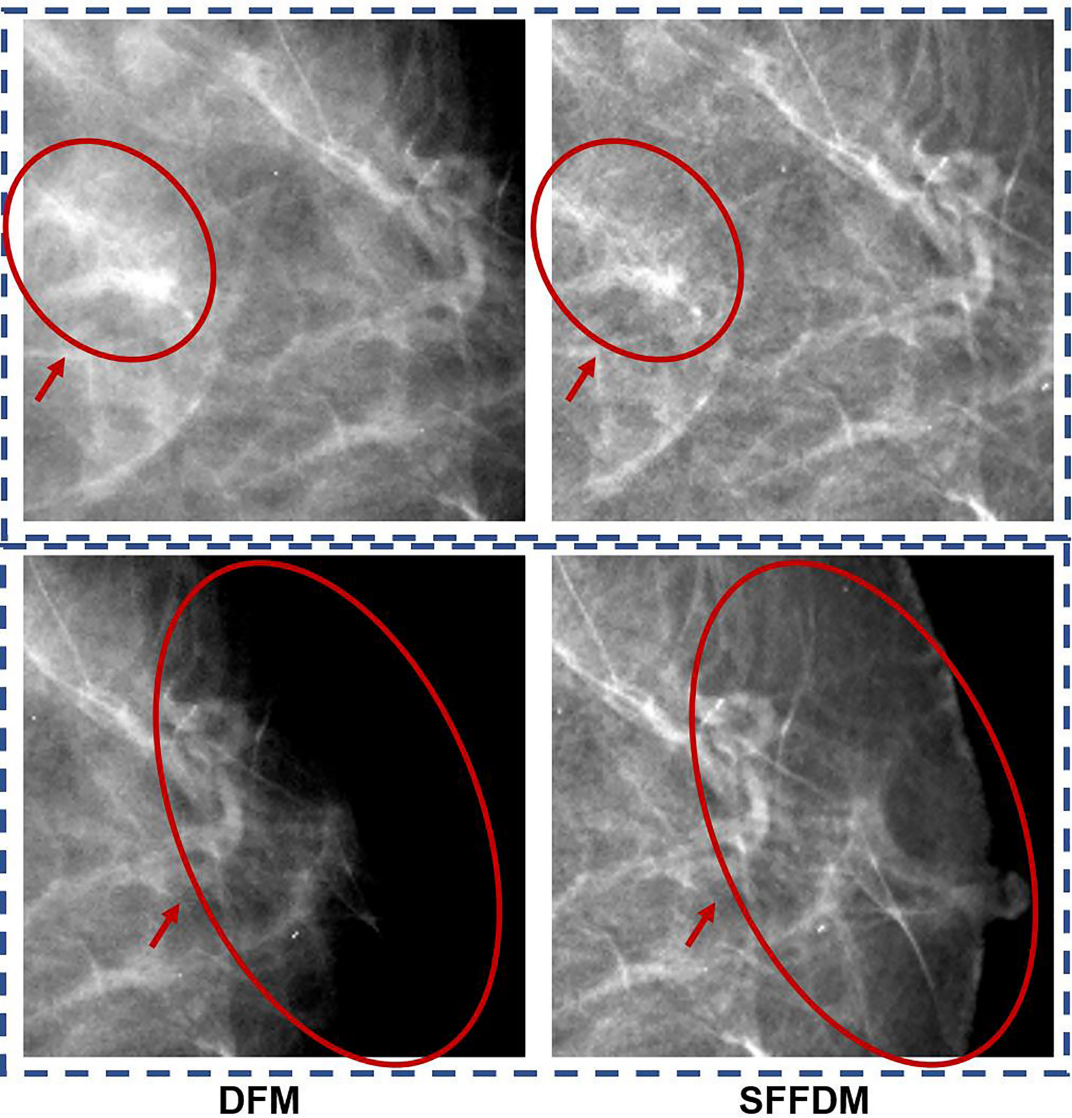
A more detailed visual comparison between DFM and SFFDM. The DFM patch in the first row of the first column showed apparent density while the SFFDM patch in the first row of the second column showed that density is due to overlapping tissue. Additionally, the nipple was barely seen in the DFM patch in the second row of the first column while it was recovered in the SFFDM patch in the DFM patch in the second row of the second column.

We first showed an example of the SFFDMs generated with HRGAN. A visual comparison of DFM and SFFDM is shown in **Figures 4**, **5**. Proper window width and window level were set in the comparison. An example of a whole high-resolution DFM and corresponding high-resolution SFFDM is shown in **Figure 4**. Two patches cropped from the DFM are illustrated in **Figure 4** and the corresponding patches cropped from the same location in SFFDM are shown in **Figure 5**.

We also showed the usefulness of HRGAN with two breast cancer screening tasks performed on dataset C. The average dice score with standard deviation across five folds for the segmentation task was shown in the first column of the table. The average AUC with standard deviation across five folds for the calcification detection task was shown in the second column of the table. As is shown in **Table 1**, the models pretrained on SFFDMs and finetuned on the training set of dataset C significantly (*p* < 10^-10^) outperformed the baseline models trained on the training set of dataset C.

**Table 1:** Experimental results of two breast cancer screening tasks.

	Dice score for the segmentation task	AUC for the calcification detection task
Baseline models	0.7012 ± 0.0102	0.8227 ± 0.0113
Finetuned models	0.7523 ± 0.0098	0.8641 ± 0.0125
p-value	<10^-10^	<10^-10^

## 4 Discussion

We proposed the HRGAN to generate detailed preserved high-resolution SFFDMs from DFMs. There was 100*μm* SFFDMs generated from 100*μm* DFMs in our experiments. Two breast cancer screening tasks including a mass segmentation task and a calcification detection task were performed to evaluate the usefulness of HRGAN. Extensive experiments showed the SFFDMs generated by HRGAN were effective to improve the performance of deep-learning-based models.

The original Cycle-GAN model was widely used in unpaired image translation tasks including translation of natural images and medical images. Despite the great power of Cycle-GAN, its performance in generating high-resolution images is limited as it failed to capture details in high-resolution images. Hence, Cycle-GAN is used for low-resolution medical images, such as CT and MR, whose resolutions are usually less than 512 × 2000 pixels but are rarely used to generate high-resolution screening mammograms whose resolutions were usually larger than 200 × 200 pixels.

To tackle the challenge of generating high-resolution medical images, we adopted the Cycle-GAN framework for unpaired image translation and supplemented our method with several techniques. A PWC training strategy was especially designed for generating SFFDM. Our pair with constraint training strategy significantly reduced inappropriate pair input and forced the model to learn proper features. In order to expand the capacity of HRGAN and capture detailed information for image translation, the U-Net-based generators were adopted. The convolutional blocks in the original U-Net were replaced by residual blocks for better capacity. The multi-scale discriminators proposed by Pix2pixHD were also adopted in our model. Besides modified network architectures, loss functions for HRGAN were also modified to capture subtle gradient changes in screening mammography. We adopted GGGAN to enhance weak edges to preserve small-scale structures.

Visual comparisons are shown in **Figures 4**, **5**. As we see in **Figure 4**, the breast region boundary is barely visible in the left DFM while the boundary is complete and clear in the right SFFDM. This clear boundary helped us locate the nipple position easily. A more detailed comparison is shown in the second row of **Figure 5**.

As was reported in Reference (9), digital mammography resulted in fewer recalls than did screen-film mammography because fortuitous positioning caused recall on screen-film mammography but not on full-field digital mammography. A detailed visual comparison in the first row of **Figure 5** showed similar results. DFM patches in the left showed apparent density while SFFDM showed that density is due to overlapping tissue. Another advantage we can observe from the detailed comparison in **Figure 5** is SFFDM has better contrast than DFM.

To quantitatively evaluate the usefulness of HRGAN, we leveraged the SFFDMs generated by HRGAN to improve the performance of deep-learning-based models when only a small number of annotated FFDMs were available. A mass segmentation task and a micro-calcification detection task were included for the evaluation. We trained the baseline models on the small FFDM dataset. For comparison, the finetuned models were first trained on SFFDMs and later finetuned on the small FFDM dataset, unlike the vanilla transfer learning (31) for medical imaging where models are usually pretrained on ImageNet (32) and finetuned on the target dataset, resulting in a large domain gap between natural images and medical images. We proposed to pretrain the breast cancer screening models on SFFDMs and finetuned FFDMs. Because the difference between SFFDMs and FFDMs is very small, the pretrained model provides a good initialization for feature extraction and is able to be finetuned to match the certain task.

One major limitation of this work is that a reader detection study was not performed. Moreover, we only performed the comparison between the baseline and finetuned models on U-Net for mass segmentation and on Vgg-16 for calcification detection. A comparison between the baseline and finetuned models on various network architectures is needed in the future. Since the major purpose of this study is not to compare different network architectures, this study did not conduct a wide investigation on various network architectures.

Additionally, our model was only trained on certain public datasets, with data acquired from limited systems. To investigate the potential capacity of the proposed method to translate DFMs to other systems such as Hologic and GE systems, more work needs to be done in the future to further quantify the cross-vendor potential of the proposed method.

## 5 Conclusion

In conclusion, the proposed HRGAN can generate high-resolution SFFDMs from DFMs. The SFFDMs were visually similar to FFDMs. Furthermore, extensive experiments showed the SFFDMs can help improve deep-learning-based model trained FFDMs.

## Data Availability Statement

The original contributions presented in the study are included in the article/supplementary material. Further inquiries can be directed to the corresponding authors.

## Author Contributions

YPZ, JW, DW, and YQZ designed the study. YPZ wrote the programs, performed data analysis, and drafted the manuscript. All authors read, discussed, approved the final manuscript, and conceived the study design.

## Funding

This work was supported in part by the NSFC under Grant 12126610, Grant 81971691, Grant 81801809, Grant 81830052, Grant 81827802, and Grant U1811461, in part by the Science and Technology Program of Guangzhou under Grant 201804020053, in part by the Department of Science and Technology of Jilin Province under Grant 20190302108GX, in part by the Construction Project of Shanghai Key Laboratory of Molecular Imaging under Grant 18DZ2260400, in part by Guangdong Province Key Laboratory of Computational Science at the Sun Yat-sen University under Grant 2020B1212060032, and in part by Guilin Technology Application and Promotion Project 20210227-9-4.

## Conflict of Interest

Author JW was employed by company Perception Vision Medical Technology Company Ltd.

The remaining authors declare that the research was conducted in the absence of any commercial or financial relationships that could be construed as a potential conflict of interest.

## Publisher’s Note

All claims expressed in this article are solely those of the authors and do not necessarily represent those of their affiliated organizations, or those of the publisher, the editors and the reviewers. Any product that may be evaluated in this article, or claim that may be made by its manufacturer, is not guaranteed or endorsed by the publisher.
